# 321. Azithromycin Use for Common Acute Respiratory Infections during a Mycoplasma pneumoniae National Surge: Results from the SHARPS-OP Benchmarking Collaborative

**DOI:** 10.1093/ofid/ofaf695.110

**Published:** 2026-01-11

**Authors:** Rana E El Feghaly, Joshua C Herigon, Matthew Kronman, Rosemary M Olivero, Sameer Patel, Michael J Smith, Ann Wirtz, Nicole M Poole, Brian R Lee

**Affiliations:** Children's Mercy Kansas City, Kansas City, MO; Children's Mercy Kansas City, Kansas City, MO; Seattle Children's Hospital / University of Washington, Seattle, WA; Helen DeVos Children's Hospital of Spectrum Health, Grand Rapids, MI; Ann and Robert H. Lurie Children's Hospital, Chicago, IL; Duke University Medical Center, Durham, NC; Children's Mercy Kansas City, Kansas City, MO; University of Colorado School of Medicine, Aurora, Colorado; Children's Mercy Kansas City, Kansas City, MO

## Abstract

**Background:**

Aside from cases of atypical pneumonia commonly caused by *Mycoplasma pneumoniae*, azithromycin is not considered first line therapy for pediatric acute respiratory infections (ARIs). Starting in late summer 2024, a nationwide surge in *M. pneumoniae* cases was reported. This study evaluated outpatient azithromycin prescriptions during that time using a nationally representative sample of institutions providing pediatric care.

**Figure 1:**
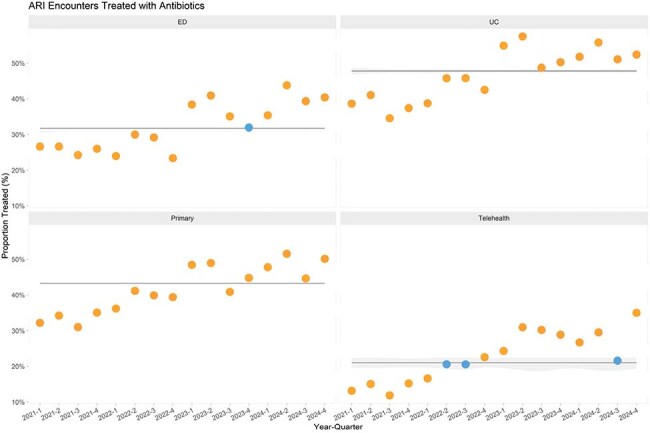
Time series of the rate of antibiotic use for ARI encounters in the different practice settings.

**Figure 2:**
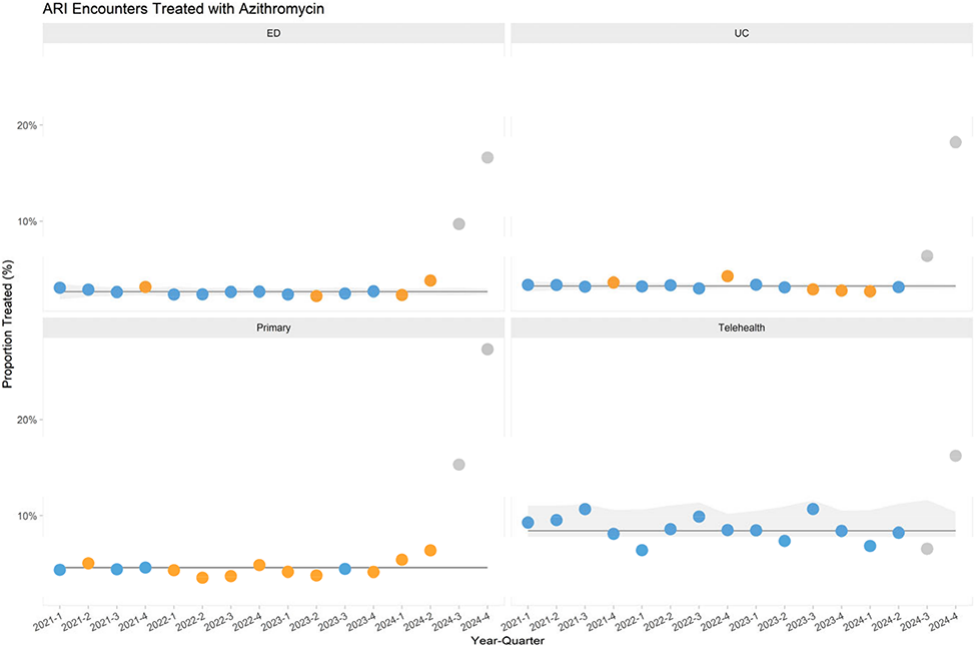
Time series of the azithromycin index in the different practice settings.

**Methods:**

Institutions from the Sharing Antimicrobial Reports for Pediatric Stewardship outpatient (SHARPS-OP) collaborative provided aggregate quarterly data January 2021 - December 2024. We collected data on all ARI encounters, and proportions of ARIs with any oral antibiotic prescription, including the proportion of amoxicillin (amoxicillin index) and azithromycin (azithromycin index) prescriptions. We stratified data by practice setting (Emergency Department [ED], Urgent Care [UC], primary care clinics [PCC], and telehealth [TH]). We compared our metrics among the different institutions and practice settings, and evaluated change over time.

**Figure 3:**
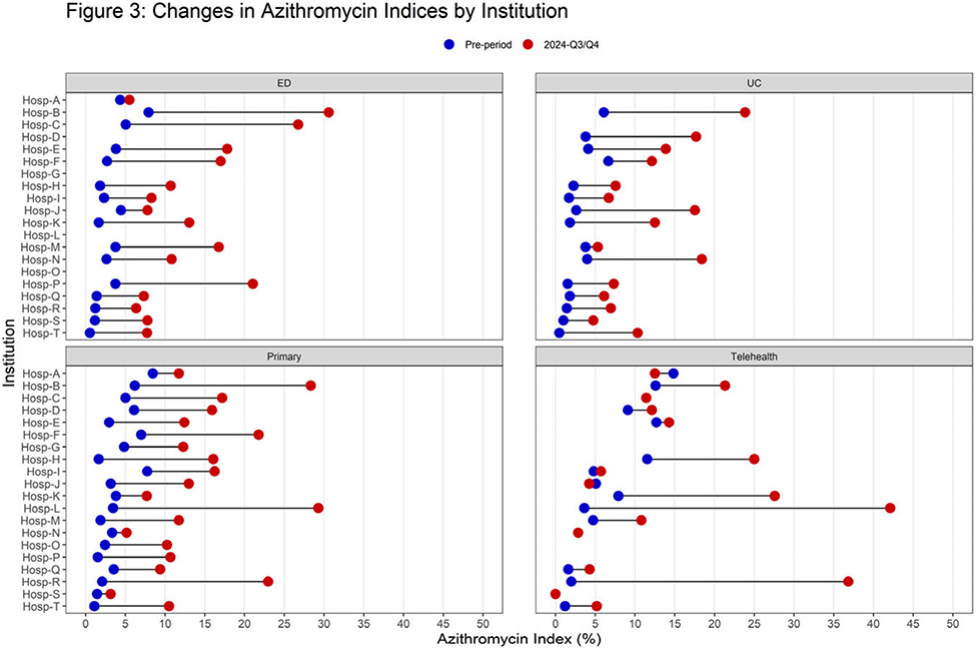
Changes in azithromycin index among the different institutions between baseline period (Q1 2021- Q2 2024), and M pneumoniae surge period (Q3-Q4 2024)

**Results:**

A total 5.8 million ARI encounters were reported across 20 institutions. Overall antibiotic prescribing for ARI encounters had been slowly increasing over the last 8 quarters (Q1 2023- Q4 2024) in all practice settings (Figure 1). We observed a small decline in amoxicillin index in Q3-Q4 2024 (67.1% prior to Q3 2024 to 61.3% Q3-Q4 2024), but a dramatic increase in azithromycin index by 16.2% (4.1% to 20.3% in Q3-4 2024). This increase was most notable in the PCC (19.0%), followed by ED (11.6%), and UCC (10.3%) (Figure 2). Figure 3 shows the variability in azithromycin index across the institutions and practice settings, with some institutions seeing no increase, while others reached an azithromycin index of over 30% in certain settings.

**Conclusion:**

Despite national guidelines advising that most *M. pneumoniae* infections do not require antibiotic therapy, azithromycin was commonly used for ARIs during the *M. pneumoniae* surge. Interestingly, azithromycin index was higher in PCC compared to the ED where severity would be expected to be higher. There was substantial variability between institutions and practice settings.

**Disclosures:**

Rana E. El Feghaly, MD, MSCI, CPHQ, Merck and Company, Inc.: Grant/Research Support|Pfizer, Inc.: Grant review panel Michael J. Smith, MD, M.S.C.E, Pfizer: Grant/Research Support

